# The Lived Experience of Racism in the Sikh Community

**DOI:** 10.1177/08862605231218225

**Published:** 2023-12-07

**Authors:** Gayle Brewer, Jatinder Singh, Minna Lyons

**Affiliations:** 1 University of Liverpool, UK; 2 Liverpool John Moores University, UK

**Keywords:** bystander intervention, lived experience, racism, religion, Sikh

## Abstract

The Sikh community may be more visible and vulnerable to racism than other religious groups, and previous research has documented the racism targeted at Sikh men and women in the United States. Relatively few studies have, however, addressed the racism experienced by Sikh communities in other countries, where racism may be less closely connected to the events of 9/11. The present study investigates the lived experience of racism in Sikh adults living in the United Kingdom. Six participants (5 male, 1 female) aged 19 to 30 years (*M* = 24.17, *SD* = 3.98) were recruited via advertisements placed on social media. Both Amritdhari Sikhs (*n* = 4) who had undertaken the Amrit Sanskar initiation ceremony or commitment and Sahajdhari Sikhs (*n* = 2) who had not undertaken the initiation participated. Semi-structured interviews were conducted (totaling 372 minutes of interview data), covering a range of subjects including personal experiences of racism and subsequent responses to the racist abuse. Interpretative Phenomenological Analysis of the interview transcripts identified five superordinate themes. These were (1) Appearance and Visibility; (2) Inevitability and Normalization; (3) Coping and Conformity (Religion as Support, Fitting In, Internalization); (4) Education and Understanding; and (5) Bystander Behavior (Experiences of Intervention, Religious Duty to Intervene, Consequences of Intervention). Findings highlight the extent to which racism occurs and the increased vulnerability of the Sikh community (e.g., appearance being the focus of racist abuse). Findings also highlight the importance of religion as a source of support and cultural pride and the significance of education and bystander behavior. Future research should further investigate these themes and introduce interventions to support the safety and well-being of members of the Sikh community experiencing racist abuse.

## Introduction

A substantial body of research has documented the prevalence and negative impact of racism (e.g., Astell-Burt et al., 2012; Harris et al., 2018). For example, racism impacts on both physical and mental health (Harris et al., 2006) to the extent that it has been conceptualized as both a traumatic experience (Ayodeji et al., 2021) and a public health crisis (Devakumar et al., 2020). It is of course important to understand the context in which racism takes place. For example, the history of the country where the racism occurs (Oikelome, 2010) and the different experiences of ethnic, religious, and cultural groups within the country (Ferdinand et al., 2015; Gorodzeisky & Semyonov, 2019) are important. The present study utilizes a qualitative approach to investigate an area that has previously received little attention, focusing on the lived experience of racism in Sikh adults living in the United Kingdom.

Sikh men and women may be more visible and vulnerable to racism than other religious groups. For example, Sikh men and women display five articles of faith known as the five K’s, including keshas (unshorn hair) and kara (an iron bracelet). In addition, Sikh men (and some Sikh women) wear turbans that are considered to be an essential part of their faith and a source of pride for the wearer (Chilana, 2005). Following the September 11th terrorist attacks, media images of Osama Bin Laden typically included a turban and beard, leading many members of the general population to associate these features with terrorism. Indeed, those wearing turbans are more likely to experience discrimination (Nadimpalli et al., 2016), and it has been argued that “the turban has transformed from a sacred piece of attire for Sikhs to a target for discriminatory conduct and an object of marginalization after 9/11” (Gohil & Sidhu, 2008 pi). As a consequence, Sikh men have reported substantial misidentification and discrimination (Ahluwalia & Pellettiere, 2010; Arora, 2013), especially in connection with extremist beliefs and perceived terrorism. Such experiences have caused considerable distress and concerns for personal safety (Ahluwalia, 2011; Arora, 2013).

Previous research has focused on racism targeted at Sikh men and women in the United States (e.g., Ahluwalia, 2011; Arora, 2013; Verma, 2006). Relatively few studies have, however, considered experiences of racism in Sikh communities in other countries, where racist abuse may be less closely associated with the events of 9/11. In one notable exception, researchers investigating the experiences of Sikh immigrants and their descendants in Ireland also document racism that includes post 9/11 references to terrorism (Jordan & Singh, 2011), suggesting a similar narrative of misidentification and abuse. The present study extends existing literature to consider personal experiences of racism (and responses to racist abuse) in those living in the United Kingdom.

One important aspect of experiencing racism is the use of individual coping strategies, which could have a major influence on mental health and self-esteem (Do et al., 2019; Ghezae et al., 2022). These coping strategies could be adaptive, maladaptive, or ambiguous and may differ depending on the level and type of racism experienced (e.g., cultural, institutional, or individual; Jacob et al., 2023). Coping strategies in response to racism have been discussed in terms of social support, gaining strength from the religion and the religious community, avoidance (e.g., appearing to be similar to others), and problem-focused coping (tackling the situation directly; e.g., Jacob et al., 2023). Coping strategies are, of course, complicated and depend on multiple factors (e.g., context, country, gender, and the racialized group an individual belongs to, e.g., Bhopal, 2022; Jaspal et al., 2021; Lewis et al., 2020 **).** There are, to date, relatively few studies investigating racism and coping strategies in the Sikh population in the United Kingdom.

There is some evidence that Sikh communities in Ireland may cope with cultural pressures by partially erasing markers of difference (e.g., changing a name) and camouflage (e.g., not wearing a turban at work; Jordan & Singh, 2011). Alternatively, faith may form an important part of the coping response. For example, Jordan and Singh (2011) reported the importance of keeping religious traditions alive, whilst Jaspal (2013) noted that for British Sikh men and women, maintaining a sense of group distinctiveness was a valued part of the response to perceived threat. Previous research has often failed to include both the experiences of those who have (Amritdhari Sikh) and have not (Sahajdhari Sikh) undertaken the Amrit Sanskar initiation ceremony or commitment. This commitment is undertaken when the individual holds the required mindset and understands the significance of the initiation (Nesbitt, 1997), suggesting that these individuals may be more likely to gain strength from their faith or religious community.

The present study investigates the lived experience of racism in British Sikh men and women using individual semi-structured interviews and Interpretative Phenomenological Analysis. This approach is especially suited to understanding the lived experience and has previously been used to investigate experiences of racism (e.g., Rehman, 2021; Tuffour, 2022).

## Method

### Participants

Six participants (5 male, 1 female) aged 19 to 30 years (*M* = 24.17, *SD* = 3.98) were recruited via advertisements placed on social media. All participants were residing in the United Kingdom and both Amritdhari (*n* = 4) and Sahajdhari (*n* = 2) Sikhs participated. See Table 1 for a summary of participant characteristics.

**Table 1.** Participant Characteristics.

Participant	Age	Gender	Amritdhari	Length of Amritdhari (Years)
1	19	M	N	Na
2	22	F	Y	<1
3	22	M	Y	5+
4	27	M	Y	5+
5	30	M	Y	5+
6	25	M	N	Na

M = male; F = female, N = no, Y = yes.

### Materials and Procedure

Those responding to the advertisement were provided with an information sheet and consent form. Individual semi-structured interviews were then conducted and recorded online. Interview length ranged from 46 to 83 minutes (*M* = 62.10, *SD* = 15.58) equating to 372 minutes of interview data. The interview schedule was prepared following established guidelines (Smith, 1995) and interviews covered a range of subjects including personal experiences of racism and subsequent responses to the racism. The research received ethical approval from the host institution (Reference: 10168) and all participants were provided with a debriefing sheet on completion of the interview. Reflecting the importance of researcher authenticity, all interviews were conducted by researcher JS, a Sikh man, who engaged in self-reflection to interrogate his positionality and the relationship between the research and researcher at all stages. Interviews were anonymized at the point of transcription.

Interviews were analyzed with Interpretative Phenomenological Analysis (Smith, 1996), an approach which allows researchers to describe, interpret, and understand the lived experience of a population and the manner in which individuals make sense of this lived experience. Therefore, themes were identified through a “bottom up” approach rather than derived from existing theory. The researchers repeatedly read the interview transcripts to aid familiarization and made notes of areas of the text of interest. Notes were formed into emergent themes, relationships between preliminary themes were identified, and these were grouped into superordinate themes and sub-themes. The analytic process was validated by discussions between authors, during which the appropriateness of each theme and sub-theme was established. Principles proposed by Smith et al. (2009) were adhered to throughout the data collection and analytic process to provide rigor and cohesion and Leininger’s (1994) six criteria (credibility, confirmability, meaning in context, recurrent patterning, saturation, and transferability) were applied to evaluate research quality.

## Results

Interpretative Phenomenological Analysis of the interview transcripts identified five superordinate themes. These were (1) Appearance and Visibility; (2) Inevitability and Normalization; (3) Coping and Conformity (Religion as Support, Fitting In, Internalization); (4) Education and Understanding; and (5) Bystander Behavior (Experiences of Intervention, Religious Duty to Intervene, Consequences of Intervention).

### Appearance and Visibility

Each participant reported that their appearance had been a focus of racist comments and behavior. For example, participants described racist comments directed at their hair or turban and attempts to remove the turban. Typical comments included “*a lot of it was you know getting your turban pulled off [awkward smile] and all these kinds of things or whatnot or people trying to touch it*” (Participant 1), “*he goes if you’re a suicide bomber you will never get away with it and all the rest of it. I think he mentioned something along the lines of that thing on your head won’t protect you or something like that*” (Participant 4), and “*about four or five of the lads made a circle around . . .and then they started saying things like you look like an alien, called me cone head and tried to touch my Jura to as they said rip it off his head*” (Participant 5).

Participants were aware that their physical appearance identified them as “different” and attracted the attention of others. For example, “*obviously I definitely look different than everybody else*” (Participant 2) and “*we do stand out a little bit, and we’re not exactly fitting into what the norm is over here. So, it does play on your mind a little bit*” (Participant 3). They were often asked questions about their appearance and the turban in particular. For example, “*it has been questioned by other people in terms of what does it mean and why do you keep your hair and whatnot. Why do you wear a turban always been questioned in that terms. Sometimes it’s been, I don’t think it has been ever questioned badly you know but most of the time it has been out of wanting to learn. As well a lot of people do want to learn why do you keep your hair, what’s the reason behind it, why do you wear a kara, why, what does your religion say about these certain things*” (Participant 1).

While their appearance attracted attention and increased the likelihood of racist abuse, participants recognized that they would also be subject to racism if they were not Sikh. As described by Participant 5, “*it would be less because like obviously Sikhi is the reason I don’t cut my hair and I look the way I do so if Sikhi wasn’t there to set out how to you know keep my appearance and live my life then I guess I wouldn’t experience those feelings of differences and the racism associated to it but I think also there would still be a level of racism there just because I’d probably to some resemble a Muslim/Hindu/foreigner and as such have all the negative experiences those groups face you know what I mean. I feel like racist people don’t really differentiate between so called foreigners you know, essentially brown people they just all group us together and label us something like terrorists or something.*”

Participants described a journey of recognizing and accepting their difference as they matured. In adulthood they not only accepted their difference but became proud of their Sikh identity and appearance, describing the positive ways in which they differed from others. For example, Participant 1 stated “*I will point someone to deas and say this is where you get your strength from, you know, be proud to be different you know you're born to stand out no one else is like us, we have a crown on our heads a crown that we carry and erm no one else carries it. We are told to act like kings and that’s because we are kings*”. Similarly, Participant 5 stated “*we have been given a distinct appearance as a gift and it is there so that you know others can see that yes that’s a Singh and for us to realize that because we have this identity we have to live by a different set of rules and we have to be held up to a higher standard than others.*”

### Inevitability and Normalization

A range of racist encounters were described, for example, “*then he starts with his racist comments and was like ‘I should kill all your people then it would be a better world’ and he pulls out a glass bottle and tries to attack me*” (Participant 6). Overall, racism was reported to be a regular and unavoidable occurrence to the extent that it was perceived as “normal” by participants. For example, “*it’s become so normal to have this type of freaking racism discrimination in your daily life. Whenever, whoever you’re going to ask I swear to God ask whatever person, I feel like everybody has, has sort of like discrimination or racism experience*” (Participant 2) and “*racism isn’t going anywhere and it is something to be expected*” (Participant 6). This experience crossed contextual boundaries, with racism experienced in education and work and perpetrated by both strangers and those known to the participant.

To some extent participants reported adjusting to racism. For example, “*you become desensitized to it*” (Participant 3). However, they were also conscious that their children were likely to experience similar racist abuse. For example, “*it is just one of those things like let’s be honest racism is not going anywhere it is all around us, it is the harsh truth but we have to accept it and on the same hand we have to realize that there are people out there who aren’t like racist and actually want to help against the battle of racism. But it does kind of make you feel sad knowing that you know probably my future kids will have to deal with it at some point and even their kids*” (Participant 4). Similarly Participant 5 stated, “*it is what it is. It’s something I have grown to accept you know what I mean, like I think it’s even something my kids are going to experience and I’ve had like talks with them just so they can prepare themselves but yeah like I said it’s just kind of something to expect, you do feel down about it to say the least, it does break you a little inside and you hear some horrifying stories and it’s like just heart breaking to be fair.*”

### Coping and Conformity

Racism had been experienced by all participants. Participants described the impact of this and the way in which they coped with racist behavior. *Religion as Support* described the way in which religion provided strength and support. *Fitting In* highlighted experiences of peer pressure and attempts to conform. *Internalization* refers to a change in beliefs to become more aligned to the beliefs of the dominant group.

#### Religion as Support

Interviews demonstrated that participants gained considerable strength from their religion. Religious beliefs, texts, and community were each important. As described by Participant 6, “*just focusing on Sikhi really like bani [holy text] is so powerful and when you realize that they can relate to all aspects of your life it is like you can tackle anything you know what I mean.*” Such comments were especially evident amongst Amritdhari participants. For example, “*I feel like our gurus is around in it you get what I am saying, but errm strength I don’t know I think I just feel more, I have more strength like let’s just say it like that because I took amrit*” (Participant 2) and “*as a Sikh especially someone who’s Amritdhari It’s very important that we have a resilient, resilient mindset. And you got to have a fatalistic mindset that you’ll get a lot of a lot of hate. Sometimes you can experience racism within, I personally I try to remind myself of our history. We’ve, there’s been so many sort of cases of oppression even currently*” (Participant 3). Furthermore, racism was sometimes conceptualized as a “test” of religious faith which provided participants with the strength to cope with racist attitudes and behavior. For example, “*if we look at Sikhi we know bro that obviously you are going to get these challenges like whilst these events were bad for me I feel like Waheguru was just testing me to see how I would react*” (Participant 4) and “*racism especially it is going to be testing how compassionate we are you know our deya [compassion]*” (Participant 5).

#### Fitting In

All participants described times when they had attempted to “fit in” with the broader social group through suppressing aspects of themselves or adopting aspects of the majority group. This process of fitting in included changes to physical appearance. For example, “I thought like getting a tattoo would help me fit in more with the gora [White person] like the other gora and probably stop all the racist sh*t because they might think I am more like them you know what I mean” (Participant 5). However, the desire to “fit in” weakened over time as participants became more confident in themselves. As described by Participant 1, “*being a young Sikh like I said you get a lot of hate and you kind of want to fit in but later on you start to realize that everyone else is different anyway and it is better that you are different than sticking with the crowd, you are born to stand out and everyone’s unique but yeah definitely in the beginning that wanting feeling to fit in because that’s what everyone has they all want to fit in er you don’t want to be that guy that is stuck out and whatnot and not included with everyone.*”

This process of Fitting In was frequently encouraged through Peer Pressure, which often influenced the decision to deviate from their faith or the external representation of this faith. For example, “*I think one of the main reasons I cut my hair was because of my gora [White person] mates at the time saying I would look better and to be honest at the time I remember thinking oh well maybe I won’t get called you know the usual raghead and so on bro but like many of my thoughts back then I regret them and genuinely if I got more into Sikhi I would have realized that much earlier*” (Participant 6). Peer pressure could, in other contexts, encourage Sikh men and women to more fully engage with their religion and display their Sikh identity to others. For example, Participant 4 described both “*surrounded by just Westernized people, from there I cut my hair and felt obliged to fit in for whatever group of people that I was within that’s why I did cut my hair*” and “*I went there with my slits in my eyebrows, my hair cut right and my earing in my ear thinking I look alright here you know and when I seen all the Singhs and that I thought I feel a bit I felt a bit ashamed here so I took it out . . .I took it out quickly because I felt a bit like nah a bit Western . . .it’s always been about trying to fit in.*”

#### Internalization

Participants described times when they had felt self-doubt either about themselves or their religion and accepted the views expressed by the dominant ethnic group. For some participants, believing these dominant views to be true and acceptable led to an internalized form of racism. For example, “*my mindset was ‘oh they are probably right maybe us Apna [people of the faith] can’t play football’. . .it is just something I thought was true so like that was one of the reasons I wanted to be more like them but also I felt like they were more popular, like the gori back then all got along and they seemed to you know what everything is . . .I would have the mindset oh they don’t want an apna [a person of the faith] they want someone better like a gora [White person]*” (Participant 5). This experience of internalized racism was more evident at a younger age before participants became confident in their beliefs and position in society. As described by Participant 4 “*as a kid I felt like maybe what they were saying was right because you know there were more of them there, so just maybe I don’t know. But when looking back I wish I could tell myself listen bro Sikhi is the way forward don’t let them tell you otherwise you get what I mean but it you know it’s one of those things you expect to happen in you know an area that is majority White.*”

### Education and Understanding

Participants often attributed racist attitudes and behavior to a lack of education or understanding. For example, “*education is key thing, when it comes to racism if there is that lack of it—education—then there is always going to be that racism. But you can always tackle it though, you just need to arm other people with knowledge about Sikhi as well*” (Participant 1) and “*because a lot of the time I feel like it’s either arrogance or they just, they don’t know any better. . . I think we should go out of our way to learn about different cultures because then things like these misconceptions and stereotypes will be avoided*” (Participant 3).

As a consequence, participants were often willing to educate others about their faith. For example, “*you have to remember like it is scary to see a kirpan if you aren’t brought up around them sort of things, so we have to understand and we have to be considerate and basically be ready to explain whatever to whoever*” (Participant 4). As stated by Participant 5 “*when I get asked about my dumalla [headwear] I am always willing to educate others on it because knowledge at the end of the day is one of our shastars [weapons] . . .because you need to have the mindset of being able to arm yourself with knowledge so think about it. If we aren’t out there spreading that knowledge that awareness who is because it isn’t taught. As well you know there’s that lack of education and for those who do know what we are there’s still a lot of misconceptions so it does fall on us to actually help and get the word out. And also, it does feel good when people ask about who you are and our history because it just shows that they want to know they are willing to learn. . .I also think that’s one way to fight against racism.*”

Education had often been effective, for example “*a lot of people they also, when they understand it they also resonate with it a lot which I’m quite happy to see*” (Participant 1). The limitations of this approach were also acknowledged. For example, “*the people who say it are stuck in their ways, so it is almost like talking to a brick wall*” (Participant 6). In particular, it could be difficult to determine whether questions were a genuine interest or a more veiled form of racist abuse. As stated by Participant 4 “*it’s good to spread as much knowledge as you can but you get those times where you have to think hmm are they asking me because they want to know are they asking me because they are being racist trying to take the mickey sort of thing. But I won’t lie most of the time it’s probably them being curious.*”

### Bystander Behavior

Responses to racist attitudes and behavior were important. Participants described their own experiences of bystander behavior (either as a witness or bystander), their perceived duty to intervene, and the potential consequences of bystander intervention.

#### Experiences of Intervention

Participants described witnessing bystander responses to racist behavior. This included numerous occasions where bystanders did not intervene. For example, “*no one reacted to it*” (Participant 2) and “*I remember—I think year 8—I was walking home and a different set of lads shouted things at me. I can’t remember what specifically they were saying but it was the usual things you get and there were other people there that pretty much did nothing I do also remember*” (Participant 5). In addition, participants described active encouragement of racism by bystanders present. For example, “*there were bystanders in [place] that egged it on, there were bystanders that didn’t do anything either*” (Participant 4).

Where they had been the target of racist abuse, participants valued interventions and support. As described by Participant 3, “*you feel supported by your fellow colleagues erm and they acknowledge that it is a thing so I think the fact that you feel supported is a big thing. . .although its not directed at them they are also experiencing that situation and they are supporting you as well it is an important thing as well.*” Participants also reported their own behavior as a bystander, either when deciding to intervene or not. For example, Participant 6 recalled, “I was walking to my mates house there were a couple of lads on bikes and they started shouting p*ki and terrorist and at first I thought it was at me and then I looked and I realized it was at a young man in front of me. So, I have turned round and just said to them don’t call him that and they were like ‘or what’ you know trying to get aggressive with me but I kept my calm and I said ‘listen you can get arrested for things like this and I’m not scared to call the police’ and *I had to bite my tongue and just not say anything.*”

#### Religious Duty to Intervene

Religion provided participants with a moral guide that influenced their own behavior as a bystander. As described by Participant 3, “*your kara is almost like a moral compass. . .sort of reminder that you have to do things with integrity, you have to do things with compassion.*” Similarly, Participant 5 stated “*when these [racist] events do happen I feel like I need to remember to keep Sikhi in my heart and use it to guide my decisions.*” Indeed, participants reported a sense of duty to support and protect others. For example, “*that’s part of being a Sikh wearing a dastar [headwear] and whatnot you should always stand up for things that aren’t right so obviously when racist comments were said or things are out of order about someone else’s religion their belief or appearance or whatnot I have always tried to step in and say something*” (Participant 1). Similarly, Participant 6 stated “*I have a dumalla [headwear] kara kirpan and I dress the way I do, it is like my uniform of a Sikh and say if a police officer was to see someone getting beat up you would expect them to help because that’s their duty it is the same with Sikhs we have that duty we need to help and stand up for others you know what I mean.*”

In addition, faith provided the strength to engage in such behavior. As described by Participant 6, “*when you really think about how you got to wear your dumalla [headwear] and the history behind it. It is like wow all that power of people who wore dumalla before you and the reasons they did. I actually remember a while ago a Sikh who used his dumalla to help a child I think that was bleeding severely until paramedics came and it is like we have been given that for a reason his strength to be courageous enough to think on his feet to not be a passive bystander and help came from the fact he has that Sikh identity do you know what I mean. I think also as well you get a sense of pride when you are out in public and that pride translates to strength and makes you carry yourself in a certain way.*” Historic examples of intervention or courageous behavior also provided the impetus for bystander behavior. For example, “*our history gives us clear examples of racism and how we have dealt with it before and our code of conduct sets out well a clear code in order to conduct yourself*” (Participant 5).

#### Consequences of Intervention

Though participants discussed their willingness to intervene where racism occurred, they also acknowledged the potential consequences of intervention. This included personal consequences such as a police record or injury. For example, “*that’s something that keeps playing on my mind and also thinking about consequences that they have on me. For example, is it really worth me getting physical with someone and them calling the police and having the implications on my future just because of a small moment of anger. There’s loads of other ways to deal with it but you really have to think is it really worth me really just losing my future*” (Participant 1). In addition, participants were concerned for the broader consequences of intervention such as the general perception and reputation of Sikh men and women. For example, Participant 4 stated “*imagine the image the media would portray or what others would think like we are representing Sikhi and so others won’t get our side of the story*” and “*how it would look to others because most racists defend their viewpoints by saying oh well this Sikh person did that so you must be the same.*”

## Discussion

Five superordinate themes were identified: (1) Appearance and Visibility; (2) Inevitability and Normalization; (3) Coping and Conformity (Religion as Support, Fitting In, Internalization); (4) Education and Understanding; and (5) Bystander Behavior (Experiences of Intervention, Religious Duty to Intervene, Consequences of Intervention).

### Appearance and Visibility

Consistent with previous research, physical appearance (and the turban in particular) was a marker of “difference” from the White majority (Singh, 2010) and often the focus of racist abuse (Nadimpalli et al., 2016). Racism targeted at those wearing turbans has increased since the 9/11 terrorist attack (Ahluwalia & Pellettiere, 2010), and this type of abuse is not confined to the United States. Indeed, Jaspal (2013) documented the attempts of British Sikhs to maintain group distinctiveness when experiencing frequent misidentification as Muslim. Racist abuse targeted at those wearing turbans requires greater attention from law enforcement and community groups. Pride in Sikh identity and appearance was also evident, especially in older adolescence and adulthood. Cultural pride has been identified as a buffer to adverse mental health outcomes when experiencing racism (Gibson et al., 2022) and has also been used in developing interventions (Stein et al., 2021). Enhancing pride in Sikh identity and culture may also form the basis of future interventions to support Sikh men and women experiencing racism in the United Kingdom.

### Inevitability and Normalization

Racism was experienced as a frequent and “normal” part of life. Indeed, research indicates that racism targeting Sikh men and women has increased (Talpur et al., 2023). Experiencing racism has a significant impact on physical and mental well-being (Ferdinand et al., 2015). For example, Nadimpalli et al. (2016) report that Sikh men and women experiencing discrimination experience poorer mental and physical health even when controlling for socioeconomic, acculturation, and social support factors. Experiences of individual racism are, of course, likely to be compounded by structural racism and the impact of this (Bansal et al., 2022; Razai et al., 2021). Interventions targeting racism are required that both reduce the incidence of racist abuse and clearly signal to the Sikh community that racist abuse is not acceptable.

### Coping and Conformity

#### Religion as Support

Previous research has documented the extent to which religion provides support (Ano & Vasconcelles, 2005), influences coping responses to racist attitudes and behavior (Beagan et al. 2012), and the impact of this on health and well-being (Reid & Dale, 2023). In particular, Ahluwalia and Pellettiere (2010) described the use of religion (e.g., prayer, teachings, principles of Sikhism) to cope with racism. This may be especially valuable for those who are unable or reluctant to access other forms of support. Future research should further investigate the supportive nature of religion and the extent to which religion may inform interventions delivered by practitioners (Ahluwalia & Alimchandani, 2013). To some extent, religion also impacted on perceptions of racist attitudes and behavior. For example, racism could be perceived as a “test” of religious faith, providing a source of strength to those experiencing it. Additional research should consider the extent to which perceiving racism as a test of faith influences responses to it.

#### Fitting In

Though research suggests that non-Sikh children with Sikh friends are less likely to display anti-Sikh attitudes, (McKenna & Francis, 2023), pressure to “fit in” with the White majority was clear in the present study. As a consequence, young people in particular may feel pressured to disengage from their religion or community (Singh, 2022). Peers have a large influence on ethnic social identity development (e.g., Jugert et al., 2020; Santos et al., 2017), and social influence from majority non-Sikh peers was evident in our sample of participants. Relatively few studies have considered the pressure to fit in experienced by Sikh children or adolescents (Gill et al., 2005) and additional research exploring the prevalence and form of this pressure should be conducted. It would be important to ensure that children and adolescents have the opportunity to interact with those from the same faith, especially for those attending schools with limited diversity.

#### Internalization

Self-doubt and internalized racism were reported by participants. This internalization was especially common at younger ages or when few members of the Sikh community were available for support and mentoring. Indeed, internalized racism develops at an early age (Elton-Chalcraft, 2009). It is important to ensure that children have opportunities to interact with others who have similar cultural experiences (e.g., dress, religion) in order to validate their perspective. Future research should consider the extent to which more recent misidentification as Muslim and accusations of terrorism have contributed to internalization of Western norms (Sian, 2013) together with the impact of internalization on mental and physical well-being (Dhillon & Ubhi, 2003).

### Education and Understanding

Participants recognized the extent to which a lack of knowledge and understanding contributed to racism (Nelson et al., 2013) which may have been exacerbated by the racialization and othering of Sikhism (Joshi, 2006). Opportunities to educate others (especially in relation to the Sikh religion) were welcomed and future research may investigate the effectiveness of this approach (McKee, 2002). Education programs incorporating lived experience may both enhance an understanding of the Sikh religion and address racism (Woods-Jaeger et al., 2023). Indeed, programs including lived experience have been employed previously to address Islamophobia (Bakali, 2016). The focus on education and understanding is consistent with research documenting compassionate responses to Sikh-based racism and a commitment to act as ambassadors of Sikhism (Ahluwalia & Pellettiere, 2010).

### Bystander Behavior

#### Experiences of Intervention

Participants reported witnessing a range of bystander behavior including failure to act and encouraging the racism. Numerous factors may impede bystander intervention (Lyons et al., 2022) and interventions should address each barrier to active intervention. Recalling their own experiences of racism, participants valued active bystander intervention. Indeed, the expectation that others will intervene mitigates the impact of the primary incident (Moschella et al., 2018; Shea et al., 2021). Where participants described their own actions as a bystander, the decision to act was often influenced by factors previously identified such as whether intervention was dangerous (Fischer et al., 2011), suggesting that further guidance on the range of actions that may assist those subjected to abuse would be beneficial.

#### Religious Duty to Intervene

In the present study, religion guided bystander behavior, with participants describing religion as a “moral compass” that gave them a duty to support and protect others. This sense of duty is especially important as failure to take responsibility is a common barrier to bystander intervention (Robinson et al., 2022). Findings are consistent with previous research (e.g., Muralidharan & Pookulangara, 2022) suggesting that those high on religiosity are more likely to intervene to support others. Indeed, research has documented the positive impact of religion and moral identity internalization on socially responsible behavior such as altruism, empathic concern, and perspective taking (Kaur, 2020). Greater recognition of this prosocial behavior, including the extent to which it is embedded within the Sikh religion (e.g., the concept of seva or self-less service) may reduce some of the racism directed at Sikh men and women (Kaur Luthra, 2021; Sohi et al., 2018).

#### Consequences

Bystander intervention may result in a range of positive and negative consequences and in the present study, participants were aware of the potential for personal harm (e.g., physical injury or arrest) or for reputational damage impacting on the wider Sikh population. Indeed, fear of reprisals is a substantial barrier to bystander intervention (Hennelly et al., 2019). Bystander training should prepare potential bystanders for the possible negative consequences of bystander intervention and highlight the range of bystander techniques available (e.g., calling authorities) that do not require direct assistance (Banyard et al., 2021).

### Limitations and Future Research

This research is not without limitations. Consistent with Interpretative Phenomenological Analysis, we recruited a relatively small homogenous sample. In particular, participants were typically male and Amritdhari. Though this approach provides a rich understanding of participant experiences, extrapolation of findings should be cautious. For example, findings cannot inform our understanding of intersectionality and future research should explore the experiences of those experiencing both racism and other forms of abuse due to a minoritized status (Cerezo et al., 2020; Lewis & Grzanka, 2016). Furthermore, research should compare the experiences of those who have and have not undertaken the Amrit Sanskar initiation ceremony or commitment, both in relation to exposure to racism and responses to it. Future studies may build upon previous research exploring the role of religion in coping behavior and approach (Morjaria-Keval, 2006; Singh, 2013; Thandi, 2011). Finally, research investigating the long-term impact of racism experienced by Sikh populations living in the United Kingdom and the effectiveness of interventions introduced to target racist abuse is required.

## Conclusion

This study adds to the paucity of literature addressing the lived experience of racism in Sikh adults living in the United Kingdom. Individual semi-structured interviews and Interpretative Phenomenological Analysis revealed five superordinate themes. These were (1) Appearance and Visibility; (2) Inevitability and Normalization; (3) Coping and Conformity (Religion as Support, Fitting In, Internalization); (4) Education and Understanding; and (5) Bystander Behavior (Experiences of Intervention, Religious Duty to Intervene, Consequences of Intervention). Future research should investigate these themes in a more diverse sample (e.g., in those with additional intersecting identities) and introduce interventions to support the safety and well-being of members of the Sikh community experiencing racist abuse.
